# Transition from neonatal to paediatric intensive care of very preterm-born children: a cohort study of children born between 2013 and 2018 in England and Wales

**DOI:** 10.1136/archdischild-2024-327457

**Published:** 2024-12-09

**Authors:** Tim J van Hasselt, Suzy Newman, Hari Krishnan Kanthimathinathan, Peter J Davis, Elizabeth S Draper, Chris Gale, Cheryl Battersby, Sarah E Seaton, The United Kingdom Neonatal Collaborative and the Paediatric Critical Care Society Study Group (PCCS-SG), Matthew Babirecki

**Affiliations:** 1 Department of Population Health Sciences, University of Leicester, Leicester, UK; 2 Parent of child with medical complexity, UK; 3 Birmingham Women’s and Children’s NHS Foundation Trust, Birmingham, UK; 4 Bristol Royal Hospital for Children, Bristol, UK; 5 Neonatal Medicine, School of Public Health, Faculty of Medicine, Imperial College London, London, UK; 6 Centre for Paediatrics and Child Health, Imperial College London, London, UK

**Keywords:** Neonatology, Paediatrics, Intensive Care Units, Paediatric, Intensive Care Units, Neonatal, Palliative Care

## Abstract

**Objective:**

Following very preterm birth, some children require ongoing intensive care after the neonatal period and transition directly from neonatal units (NNUs) to paediatric intensive care units (PICUs) around term-corrected age.

We aimed to understand, at a national level, characteristics and outcomes of children born very preterm who transitioned directly from NNUs to PICUs.

**Design:**

Retrospective cohort study, using data linkage of National Neonatal Research Database, Paediatric Intensive Care Audit Network and Office for National Statistics datasets.

**Setting:**

All NNUs and PICUs in England and Wales.

**Patients:**

Children born <32 gestational weeks between 1 January 2013 and 31 December 2018, admitted to NNUs, and who transitioned directly to PICU without return to NNU at ≥36 weeks corrected gestation age were included.

**Main outcome measures:**

Mortality, length of PICU stay, invasive ventilation in PICU (including via tracheostomy), PICU readmission until 2 years of age.

**Results:**

Direct NNU-to-PICU transitions occurred in 276 babies during the study period. An increasing yearly trend was observed: 36 transitions of babies born in 2013, 65 in 2018.

Of this cohort, 22% of children died before their second birthday, 59% of survivors had ≥1 PICU readmission, 33% of children had long stays in PICU (≥28 days) and 25% received tracheostomy ventilation.

**Conclusions:**

An increasing number of very preterm children require ongoing intensive care at the end of their neonatal stay, with high rates of mortality and morbidity. Multidisciplinary involvement and planning around the time of transition from NNU to PICU, informed by national guidance, may be beneficial.

**WHAT IS ALREADY KNOWN ON THIS TOPIC**

- Some very preterm-born children may require ongoing intensive care after the neonatal period and transition directly from neonatal care to paediatric intensive care units (PICU), without going home.
- There are no national population-level data describing the number of children transitioning directly from neonatal units to PICUs as they approach term corrected age.
- We aimed to characterise this important group of children with complex needs, including those with severe bronchopulmonary dysplasia requiring long-term positive pressure ventilation.

**WHAT THIS STUDY ADDS**

- We used linkage of the National Neonatal Research Database and the Paediatric Intensive Care Audit Network to examine neonatal-to-PICU transitions.
- We identified 276 children born <32 weeks and transitioned from 36 weeks corrected gestational age in England and Wales between 2013 and 2018.
- 22% died before their second birthday, a third had long stays in PICU of 28 days or more and a quarter received tracheostomy ventilation.

**HOW THIS STUDY MIGHT AFFECT RESEARCH, PRACTICE OR POLICY**

- These data highlight the high rates of mortality and morbidity in this population; clinicians could consider whether there may be benefit from early involvement of medical complexity teams and parallel planning.
- These results provide evidence to inform the development of a neonatal-to-PICU transition pathway, to inform service provision and identify resource needs.
- These data will aid in communication with families during this prolonged period of intensive care and challenging transition.

## Background

 With the increasing survival of babies born very preterm (<32 weeks gestational age), more children are surviving with ongoing morbidity.^1^ Some children require ongoing intensive care beyond 44 weeks corrected gestational age (CGA), after which care within neonatal units (NNUs) may not be the most appropriate location of care and is not routinely funded in the UK.^2^ Such babies may therefore be transitioned directly from NNU to paediatric intensive care units (PICUs). Reasons for this include severe bronchopulmonary dysplasia (BPD), potential need for long-term ventilation (LTV)^3^ or morbidity requiring ongoing hospitalisation arising from conditions such as necrotising enterocolitis (NEC) or intraventricular haemorrhage (IVH).^4^

We aimed to understand the characteristics of very preterm children transitioned from neonatal to PICUs in England and Wales from 36 weeks CGA, using national linked data, and to describe the subsequent course and outcomes in PICU.

## Methods

The National Neonatal Research Database (NNRD) provided data for all children born between 22 and 31 completed weeks gestational age from 1 January 2013 to 31 December 2018 and admitted for neonatal care in England and Wales. Following linkage to data from the Paediatric Intensive Care Audit Network (PICANet), we identified PICU admissions in England and Wales before the age of two years (chronological age, uncorrected). The duration of follow-up was based on the aim to examine a contemporary neonatal population, as the majority of children admitted to PICU are under two years of age.^5^ Based on input from our expert multidisciplinary clinical advisory group (online supplemental table 1), transition from neonatal-to-PICU care was defined as a recorded PICU admission occurring within 24 hours of neonatal discharge, without return to neonatal care, occurring from≥36+0 weeks+days CGA, where the final neonatal discharge destination was recorded as ongoing care rather than discharge home.

The NNRD captures demographic and clinical data for all admissions to NNUs in England and Wales since 2013.^6^ PICANet collects demographic and clinical data for all PICU admissions in the UK and Ireland, with complete coverage for England and Wales from 2003.^5^ Both datasets undergo verification and data cleaning. National Health Service (NHS) Digital (now NHS England) performed data linkage using personal identifiers (NHS number present in >99% of children, date of birth, surname, postcode) and provided pseudonymised linked data.^7^

We performed descriptive statistics on maternal and neonatal characteristics from NNRD data, and on PICU care delivery and outcomes from PICANet. Categorical variables were described as frequencies and percentages, and continuous variables as mean and SD if normally distributed or median and IQR if not. Birth weights and weights on transition≥3 SD from the median^8^ were replaced with missing values but not excluded. Small for gestational age was defined as <10th centile birth weight for gestation and sex using existing thresholds.^9 10^

The NNRD provided data for congenital anomalies of any severity, and for severe congenital anomalies (life-limiting or requiring surgical intervention), we applied the Helenius *et al* definition.^11^ We also examined: retinopathy of prematurity≥stage 3 or treated; BPD requiring oxygen or respiratory support from 36 weeks CGA; neonatal brain injury, including grade III/IV IVH, periventricular leukomalacia, hydrocephalus and meningitis^12^; severe NEC requiring surgery^13^; and pulmonary hypertension, excluding primary pulmonary hypertension of the newborn. NNRD care records provided CGA, weight, receipt of parenteral nutrition (PN) and respiratory support on the final day of neonatal care.

PICANet provided outcomes data for length of PICU stay, PICU readmission, tracheostomy ventilation within PICU and death within PICU. Office for National Statistics data for mortality in any location before the age of 2 years were also linked. PICANet Read Codes of primary diagnoses and morbidities across admissions were cross-mapped to International Classification of Diseases (ICD-10) codes^14^ to allow application of the classification of paediatric chronic disease.^15^ We created box-and-whisker plots of gestation at birth and CGA at transition by PICU primary diagnosis category.

This research project benefited from extensive input including study design and interpretation of results, from families invited to online patient and public involvement (PPI) meetings via Bliss (the charity for preterm and sick babies), and our parent author (SN).

## Results

**Figure 1:**
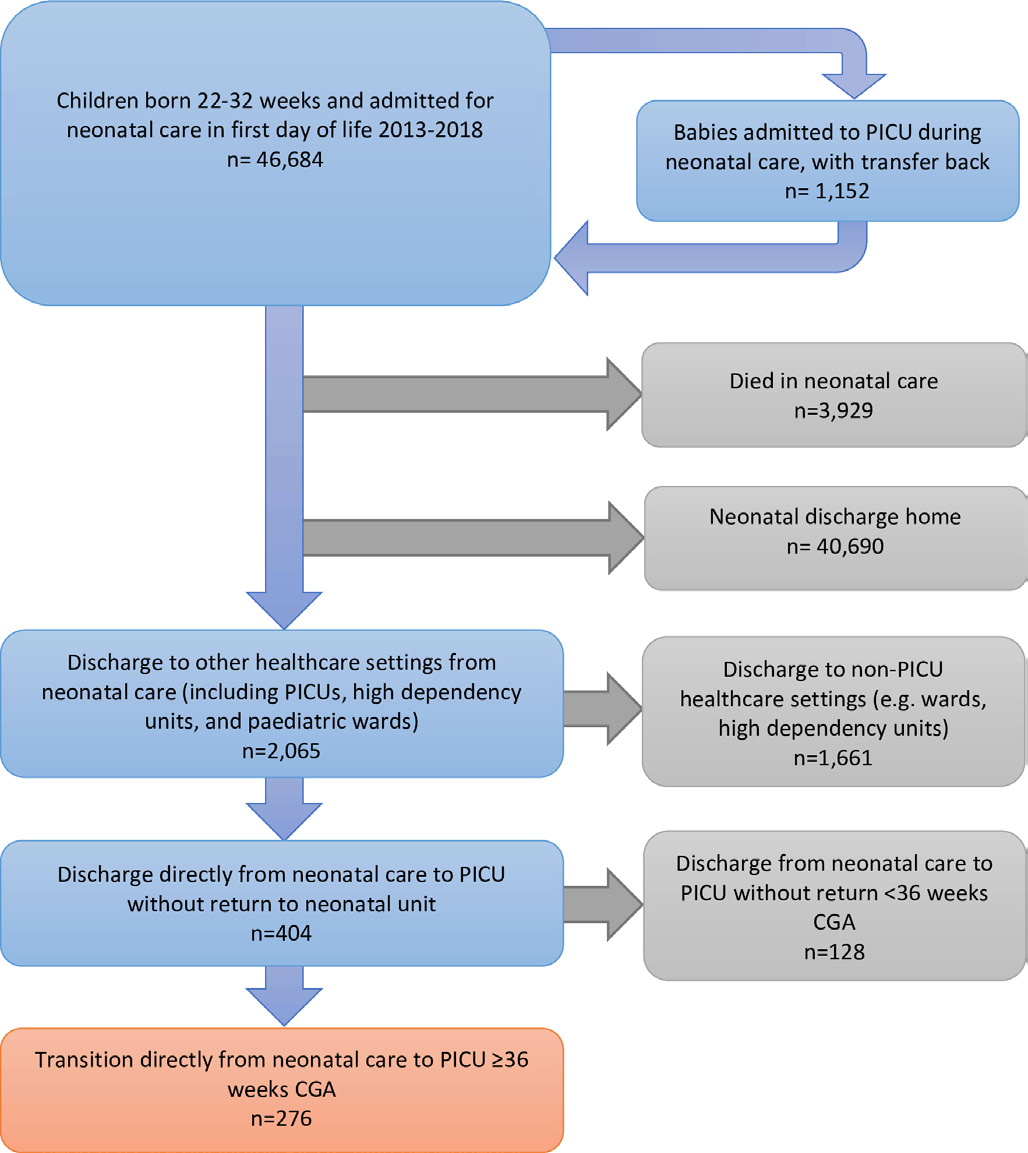
Study flowchart. CGA, corrected gestational age; PICU, paediatric intensive care unit.

There were 46 684 babies born between 22 and 31 weeks and admitted to NNUs in England and Wales from 2013 to 2018 in the first day of life (1.1% of 4 119 983 live births in this period).^16^ There were 3929 children (8.4%) who died in NNUs, and 40 690 children (87.2%) discharged home—this latter group were described previously.^17^ There were 2065 babies discharged to other healthcare settings, of whom 276 transitioned to PICU from 36 weeks CGA (0.6% of neonatal admissions born<32 weeks) (figure 1).

**Figure 2:**
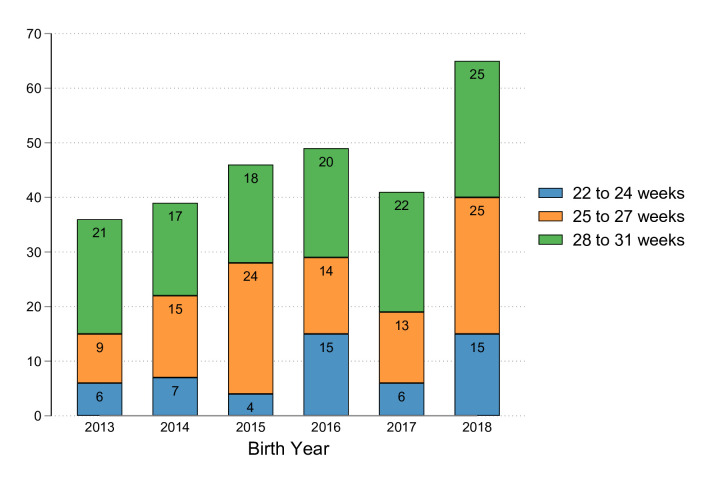
Number of children transitioned from neonatal-to-paediatric intensive care unit≥36 weeks corrected gestational age, by year and gestation at birth.

The number of very preterm neonatal admissions decreased from 7823 in 2013 to 7416 in 2018, matching falling birth rates.^16^ Despite this, the number of babies transitioned to PICU≥36 weeks CGA increased from 36 babies born in 2013 (0.5% of neonatal admissions) to 65 born in 2018 (0.9% of neonatal admissions) (table 1 and figure 2). There were similar increases in PICU care received, as measured by PICU bed days from transfer until the age of 2, from 1389 days for children born in 2013 to 3081 for those born in 2018 (online supplemental figure 1). Due to the relatively small numbers, the number of bed days per individual child varied from year to year, making comparison difficult.

**Table 1:** Birth and neonatal characteristics of the cohort born 2013–2018, <32 weeks

		Children born <32 weeks and admitted to neonatal unit, not transitioned to PICU≥36 weeks CGA		Neonatal-to-PICU transitions≥36 weeks CGA	
		n	%	n	%
Total (% by row)		46 408	99.4	276	0.6
Birth year (% by row)	2013	7787	99.5	36	0.5
	2014	7658	99.5	39	0.5
	2015	7925	99.4	46	0.6
	2016	7886	99.4	49	0.6
	2017	7801	99.5	41	0.5
	2018	7351	99.1	65	0.9
Gestation at birth (% by row)	<24	1387	98.7	18	1.3
	24	2427	98.6	35	1.4
	25	2821	99.1	25	0.9
	26	3464	98.9	39	1.1
	27	4293	99.2	36	0.8
	28	5673	99.4	33	0.6
	29	6506	99.5	35	0.5
	30	8529	99.7	25	0.3
	31	11 308	99.7	30	0.3
Sex (% by column)	Male	25 369	54.7	183	66.3
	Female	21 008	45.3	93	33.7
	Missing	31	0.1	0	
Birth weight (g)	Mean (SD)	1204 (378.0)		952 (384)	
	SGA (% by column)	3919	8.5	61	22.5
	Missing	280	0.6	5	1.8
Invasive mechanical ventilation in neonatal care (% by column)	Received	31 632	68.1	266	96.4
	Total days, median (IQR)	2 (0–6)		33 (10–60)	
	% neonatal days ventilated, median (IQR)	3.6 (0–12.0)		26.1 (11.9–45.6)	
BPD (% by column)	None	29 381	63.3	30	10.9
	Present	13 118	28.3	242	87.7
	Died before 36 weeks	3638	7.8	–	
	Missing	271	0.6	4	1.5
Congenital anomaly (% by column)	Any	1146	2.5	51	18.5
	Severe*	363	0.8	37	13.4
Severe NEC (% by column)	Present	1622	3.5	42	15.2
Brain injury (% by column)	Present	3785	8.2	61	22.1
ROP≥stage 3 or treated (% by column)	Present	2889	6.2	66	23.9
	Missing	4631	10.0	8	2.9
Pulmonary hypertension diagnosed in neonatal care (% by column)		463	1.0	21	7.6
Tracheostomy during neonatal care (% by column)	Present	300	0.7	24	8.7
Corrected gestational age on day of transition (% by column)	Median, IQR	–		44.6 (40.3–49.9)	
	Range	–		36–68	
	Transition≥44 weeks CGA			149	54.0
Weight on day of transition (g)	Median (IQR)	–	–	3096 (2513–3896)	
	Missing	–	–	23	8.3
Respiratory support on day of transition (% by column)	Invasive ventilation	–	–	103	37.3
	NIV including high flow oxygen	–	–	102	37.0
	None/low flow oxygen	–	–	70	25.4
	Missing	–	–	1	1.1
PN use on day of transition (% by column)	Received	–	–	39	14.1
	Missing	–	**–**	8	2.9

* Helenius et al definition, *BMJ* 2019

BPD, bronchopulmonary dysplasia (requirement for oxygen or respiratory support at 36 weeks CGA); CGA, corrected gestational age; NIV, non-invasive ventilation; PICU, paediatric intensive care unit; PN, parenteral nutrition; ROP, retinopathy of prematurity; Severe NEC, necrotising enterocolitis requiring surgery; SGA, small for gestational age (<10th centile).

### Characteristics of children transitioned

Babies transitioned to PICU were born earlier than those who were not (median 27 vs 29 weeks), had lower birth weight (mean 952 g vs 1204 g) and more were male (66.3% vs 54.7%) (table 1). Other perinatal characteristics were broadly similar between groups (online supplemental table 2). A greater percentage of children who transitioned to PICU received invasive ventilation during neonatal care (96.4% vs 68.1%), had BPD (87.7% vs 28.3%) or had tracheostomies (8.7% vs 0.7%) compared those not transitioned (table 1). The prevalence of other significant morbidities such as severe NEC (15.2% vs 3.5%) and brain injury (22.1% vs 8.2%) was also higher. Moreover, 18.5% had congenital anomalies (including 37, 13.4%, with severe congenital anomalies, of whom 30, 10.9%, had congenital cardiac disease), compared with 2.5% of those not transitioned.

### Characteristics of transition to PICU

**Figure 3:**
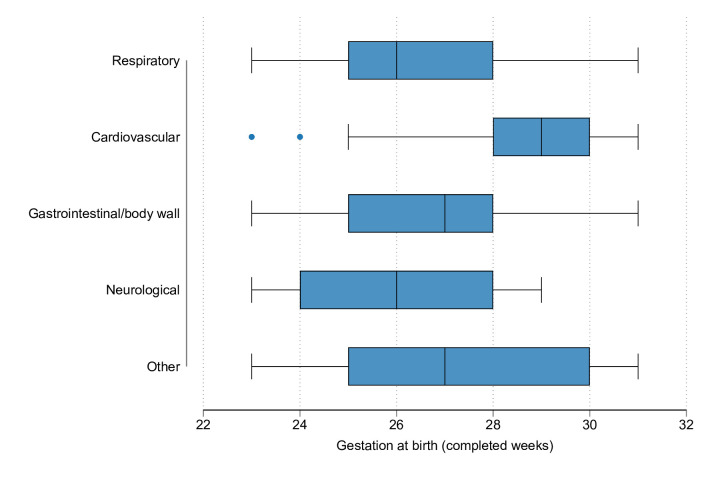
Box-and-whisker plot of gestational age at birth by primary diagnosis category on transition to paediatric intensive care unit. Note: ‘body wall’ diagnoses include congenital abdominal wall defects, congenital diaphragmatic hernia, inguinal hernia and codes for open laparotomy procedures.

The most frequent PICU primary admission diagnosis categories for transitions were respiratory (44.2%) followed by cardiovascular (21.0%) (table 2). Children with respiratory admissions also comprised the majority of PICU bed days (7512/13 834 bed days, 54.3%, (online supplemental figure 2). 71 admissions were for surgery (26%). Gestation at birth was higher for children transitioned for cardiovascular diagnoses (median 29 weeks), compared with respiratory diagnoses (median 26 weeks) (figure 3).

**Table 2:** PICU admission characteristics and outcomes

		Neonatal-to-PICU transitions≥36 weeks CGA	
		n=276	
		n	%
PICU primary admission diagnostic category on transition	Respiratory	122	44.2
	Cardiovascular	58	21.0
	Gastrointestinal/body wall	51	18.5
	Neurological	8	2.9
	Other	35	12.7
	Missing	2	0.7
Presence of pulmonary hypertension in any PICU admission		44	15.9
Presence of chronic conditions across organ systems across any PICU admission	0	46	16.7
	1	99	35.9
	2	62	22.5
	>2	69	25.0
	Any chronic respiratory condition	116	42.0
	Any chronic cardiac condition	154	55.8
	Any chronic GI/metabolic condition	104	37.7
	Any chronic neurological condition	55	19.9
Invasive ventilation during PICU stay after transition	Received	194	70.3
	Days, median (IQR)	6 (0–18)	
	Missing	17	6.2
Tracheostomy ventilation during PICU transition admission	Received	46	16.7
	Missing	17	6.2
Tracheostomy ventilation during any PICU admission	Received	70	25.4
	Missing	15	5.4
Length of stay on PICU transition admission, days	Median for all children (IQR)	13 (5–29)	
	Median for children who died on PICU transition admission (IQR)	24.5 (11.5–44)	
	Median for survivors (IQR)	11 (5–27)	
	Children with long stay ≥28 days	71	25.7
Length of stay including all subsequent admissions	Days, median (IQR)	23.5 (9–62)	
	Any long stay ≥28 days	92	33.3
Number of PICU readmissions per child occurring after transition	0	132	47.8
	1	52	18.8
	2	28	10.1
	3	18	6.5
	>3	46	16.7
Death on transition admission	Died	32	11.6
Death on any PICU admission before 2 years of age	Died	47	17.0
Death before 2 years of age	Died	60	21.7

CGA, corrected gestational age; GI, Gastrointestinal; PICU, paediatric intensive care unit.

The median CGA at transition was 44.6 weeks (range 36–68, IQR 40.3–49.9) (table 1 and online supplemental figure 3). CGA on transition was higher (45.9 weeks, IQR 40.9–50.3) for the 103 children (37.3%) who received invasive ventilation or the 102 children (37.0%) who received non-invasive ventilation (NIV) (45.6 weeks, IQR 41.0–50.6) on their last day of neonatal care, compared with the 70 children (25.4%) who were self-ventilating in air or low-flow oxygen (42.3 weeks, IQR 38.9–46.7).

There were 39 children (14.1%) who received PN on the day of transition, which may suggest short bowel, intestinal failure or increased nutritional requirements.

### Outcomes within PICU

Most children (n=230, 83.3%) had at least one chronic condition recorded across any PICU admission, and 131 (47.4%) had chronic conditions across more than one organ system (table 2). Following transition, 70.3% (n=194) of children received invasive ventilation. Duration of invasive ventilation was longer for children who were ventilated on the NNU at the time of transition (median 14.5 days, IQR 7–39) compared with those who received NIV (median 2 days, IQR 0–13) or no positive pressure support (median 2 days, IQR 0–6).

There were 46 children (17%) who received tracheostomy ventilation following transition to PICU, with a median duration of 44 days (IQR 22–39 days) of invasive ventilation (by endotracheal or tracheostomy tube). This percentage increased to 25% (n=70) of children across any PICU admission from transition to 2 years of age. There were 55 children (19.9%) with a diagnosis of pulmonary hypertension recorded in NNU or PICU, 20 of whom received tracheostomy ventilation.

Following transition, 32 children died (11.6%) during the initial PICU admission (table 2). Among survivors, few children were discharged to hospices (n<5, rounded for non-disclosure). Mortality until 2 years of age was 17.0% within PICU and 21.7% in any location. Among high-risk subgroups, 8 of the 37 children (22%) with severe congenital anomalies died before their second birthday, of whom 7/30 (23%) children with cardiac conditions died; as did 18 (26%) of children who received tracheostomy ventilation; 17 (31%) of those with pulmonary hypertension; and 8 (21%) of those receiving PN on the day of transition. More children who were invasively ventilated on the final day of neonatal care died (33/103, 32.0%) compared with those receiving NIV (19/102, 18.6%) or not receiving positive pressure support (8/70, 11.4%).

The median length of transition PICU stay for survivors was 11 days (IQR 5–27); however, for those who died it was 24.5 days (IQR 11.5–44) (table 2). One-third of children (n=92) had at least one PICU long stay (≥28 days), the majority of these were during the transition admission (n=71, 25.7%). Of 244 survivors following transition, 144 (59%) were readmitted to PICU before the age of 2.

## Discussion

Despite improvements in neonatal care and a decline in birth rate, a small but increasing number of very preterm-born children (40–50 per year) transition directly from NNUs to PICUs. These children have a high risk of mortality and considerable healthcare needs, including prolonged PICU stays and PICU readmission, and a quarter receive tracheostomy ventilation. While the predominant reason for transition was respiratory disease, many had multimorbidity.

While the percentage of preterm babies admitted to PICU during the birth hospitalisation has been described (5.5% for those born <28 weeks, 1.7% for those born 28–31 weeks),^18^ we are not aware of any study that uses national data to examine preterm-born children transitioned from NNU to PICU for ongoing intensive care.

The majority of children transitioned (88%) had BPD. The recent British Paediatric Surveillance Unit (BPSU) study of life-threatening BPD identified 153 babies in 2017–2018 who were dependent on positive pressure support or pulmonary vasodilators at 38 weeks CGA,^19^ 34% had pulmonary hypertension and 16% died before the age of 1 year. Mortality in our study was similar, and a considerable percentage (20%) had pulmonary hypertension. However, many babies in the BPSU study may not have required intensive care by 44 weeks CGA, so were discharged elsewhere, and hence not captured in our study.

A 2019 UK census of 2383 children requiring LTV identified 38 admitted to acute wards or PICUs.^3^ Gestation at birth was not reported, and only 65 children required LTV due to BPD; however, other complications of prematurity (airway obstruction or neurodisability) may have contributed. While PICANet does not report LTV, we found one quarter of children transitioned received tracheostomy ventilation, suggestive of LTV. However, we could not examine children establishing LTV within high-dependency units, as PICANet did not collect this during the study period.

We observed similar mortality rates to those observed among extremely preterm-born children with PICU long stays (23.8%),^20^ or children with medical complexity and multiple PICU admissions (16%).^21^ These figures are much higher than the overall mortality rate in PICU for either the general UK PICU population (3.5%)^5^ or for very preterm-born children admitted to PICU after neonatal discharge home (2.4%).^17^ The length of PICU stay was also higher for the children who eventually died in PICU. However, the majority of children survived following PICU admission. Direct transition to a hospice was extremely rare, in keeping with data from PICANet reports, and the majority of deaths took place in PICU following admission.^5^ Our findings support the recommendations from the recently published BAPM document 'Recognising uncertainty: an integrated framework for palliative care in perinatal medicine’ which advocate for early parallel planning for complex babies with an uncertain future.^22^ This may still involve transitioning care to PICU in some circumstances, whereas alternative locations such as a hospice, home or delivery of palliative care within the NNU may be appropriate in others. Regardless, the data from this study showing one in five children transitioned to PICU did not survive beyond 2 years of age provides data to underpin discussions around parallel planning and palliative care with families.

Our parent author (SN) experienced transition from neonatal to PICU as her son was born very preterm with congenital heart disease. She observed that the environment of PICU was different from the NNU: faster paced, noisier, with more uncertainty and frequent medical emergencies. This resulted in a frightening and overwhelming experience for the family. In addition, differences in routines and treatment protocols between NNU teams and PICU teams were disconcerting and provoked uncertainty. The family felt that the delivery of care was more consistent and they were better supported when specialist neonatal nurses were introduced within PICU, after discussions between the family and the clinical team. As the constant presence by her child, SN became an expert parent and found herself feeling less helpless. She was able to help with nursing care, and as she noticed changes in her son’s condition, her subsequent suggestions to the clinical team were acted on. SN’s experiences are not unique, they align with the previous literature describing the challenges in communication between healthcare teams and families of children requiring extended intensive care, and the importance of appropriately preparing families for the transition between two quite different clinical environments.^23 24^ Collaborative delivery of care between families and healthcare professionals may enable parents and guardians to contribute to optimal and consistent care for their child.^22^

### Policy and research implications

Given the well-described challenges in transition of care between different healthcare settings,^24^ and the parental experiences described here and in the literature, the introduction of a well-planned transition process appears prudent. This potentially would involve the family, different members from the neonatal and PICU teams, as well as other subspecialties where relevant early within the transition process, including medical complexity teams, to improve continuity of care. Although regional guidelines currently exist in some places, this is not universal. We anticipate that the recent announcement of the British Association of Perinatal Medicine Working Group on Transition to Paediatrics will lead to introduction of a consistent and streamlined transition process between NNU and PICU. This could follow the example of the UK guidelines for transition from paediatric to adult intensive care services, released jointly from the Paediatric Critical Care Society and Intensive Care Society.^25^

Due to risk of neurodevelopmental impairment, these children should receive neonatal clinic follow-up.^26^ Similarly, clinical teams should ensure that respiratory syncytial virus prophylaxis is provided to high-risk children, despite transition.^27^

Further quantitative and qualitative research is required to better understand this population, potentially applying the Post Intensive Care Syndrome in Children framework,^28^ and investigate interventions to improve outcomes, experience and quality of life of children and families.

### Strengths and limitations

The use of linked large datasets allowed identification and description of babies transitioned from NNUs for ongoing intensive care at a national level over 6 years, including perinatal factors and later outcomes. Over 99% of children had NHS numbers, ensuring a high proportion of reliable linkage. Our study also benefited from PPI input from families with lived experience, throughout the research process and a parent author, to provide context for our qualitative results.

Routinely collected data may be subject to input error or omission, for example, underestimation of multimorbidity if comorbidities were not completely recorded. Moreover, the classification of chronic disease in children was not developed for the very preterm-born population, therefore less accurately describes neonatal multimorbidity. We could not describe the outcomes of LTV and neurodevelopmental follow-up from available data. Due to the relatively small numbers, we were limited to descriptive statistical analysis.

We used an inclusion threshold of 36 weeks CGA at transition, which excluded younger babies transferred to PICU acutely (eg, with severe NEC) who then died. Analysis of this group would be complex due to geographical variation in service provision in the UK.^5^

During the study period PICANet did not collect data from high dependency units; however, this has subsequently commenced, and future linkage studies could describe these transitions. Further studies, such as NeoWONDER,^29^ could also examine the growing population of children born at 22–23 weeks, after changes in UK guidance on neonatal resuscitation were released in 2019,^30^ as this group may contribute further to the increasing prevalence of BPD and need for prolonged critical care. We did not have data on the nature of hospital or regional-level neonatal-to-PICU transition pathways or guidelines and so were unable to compare this to the outcomes observed.

## Conclusions

A relatively small but increasing number of very preterm children in England and Wales require ongoing intensive care at the end of their neonatal stay, and transition directly to PICU. This population of children are medically complex and require considerable and sustained PICU resource. National guidance should inform transition of care for this complex group, with co-ordinated multidisciplinary involvement. More research is needed to better understand babies with ongoing complex medical needs and multimorbidity after very preterm birth.

## Supplementary material

10.1136/archdischild-2024-327457 online supplemental file 1

## Data Availability

Data may be obtained from a third party and are not publicly available.
