# Supplementary material for: Transition from neonatal to paediatric intensive care of very preterm-born children: a cohort study of children born between 2013 and 2018 in England and Wales
Source: Arch Dis Child Fetal Neonatal Ed. 2024 Dec 9;110(4):e327457. doi: 10.1136/archdischild-2024-327457 (PMC12229064; doi:10.1136/archdischild-2024-327457)

Supplementary Table 1 – Clinical advisory group

NAME	ROLE	SITE
Dr Amitava Sur	Neonatal consultant	East Lancashire Hospitals NHS Trust
Dr Cara Morgan	Paediatric cardiology trainee	Royal Brompton & Harefield hospitals
Dr Isobel Brookes	Respiratory consultant	Birmingham Women's and Children's NHS Foundation Trust
Dr Katie Prentice	PICU consultant	NHS Lothian
Dr Patrick Davies	PICU consultant	Nottingham University Hospitals NHS Trust
Dr Paula Evram	PICU consultant	Sheffield Children's NHS Foundation Trust
Dr Peter Davis	PICU consultant	University Hospitals Bristol NHS Foundation Trust
Dr Reena Bhatt	Neonatal consultant, academic with respiratory and long-term ventilation interest	University College London Hospitals NHS Foundation Trust
Dr Rum Thomas	PICU consultant	Sheffield Children's NHS Foundation Trust
Dr Sarah Sparrow	Neonatal consultant	Royal Hospital for Children Glasgow
Dr T'ng Chang Kwok	Neonatal GRID trainee, research fellow	University of Nottingham

Supplementary Table 2 – Maternal and Birth characteristics

		Children born <32 weeks and admitted to neonatal unit, not transitioned to PICU ≥36 weeks CGA		Neonatal to PICU transitions ≥36 weeks CGA	
		**n**	**% (by column)**	**n**	**% (by column)**
**Total**		**46,408**		**276**	
**Multiple**	**Single**	34,333	74.0	216	78.3
	**Multiple**	12,075	26.0	60	21.7
**Antenatal steroids**	**None**	2,617	5.6	9	3.3
	**Complete**	32,271	69.5	206	74.6
	**Incomplete**	8,506	18.3	33	12.0
	Missing	*3,014*	*6.5*	*28*	*10.1*
**Maternal ethnicity**	**White British**	25,343	54.6	134	48.6
	**Other ethnic group**	819	1.8	22	8.0
	**Other white**	3,636	7.8	20	7.3
	**Black**	3,601	7.8	33	12.0
	**Pakistani**	2,007	4.3	25	9.1
	Missing	7,083	15.3	*42*	*15.2*
**IMD (deciles, 1=most deprived, 10=least deprived)**	**Median (IQR)**	4 (2 to 7)		4 (2 to 6)	
	Missing	*9,445*	*20.4*	48	17.4

Supplementary Figure 1 – total annual PICU bed days utilised between transition from neonatal care to PICU until two years of age, by gestation and birth year

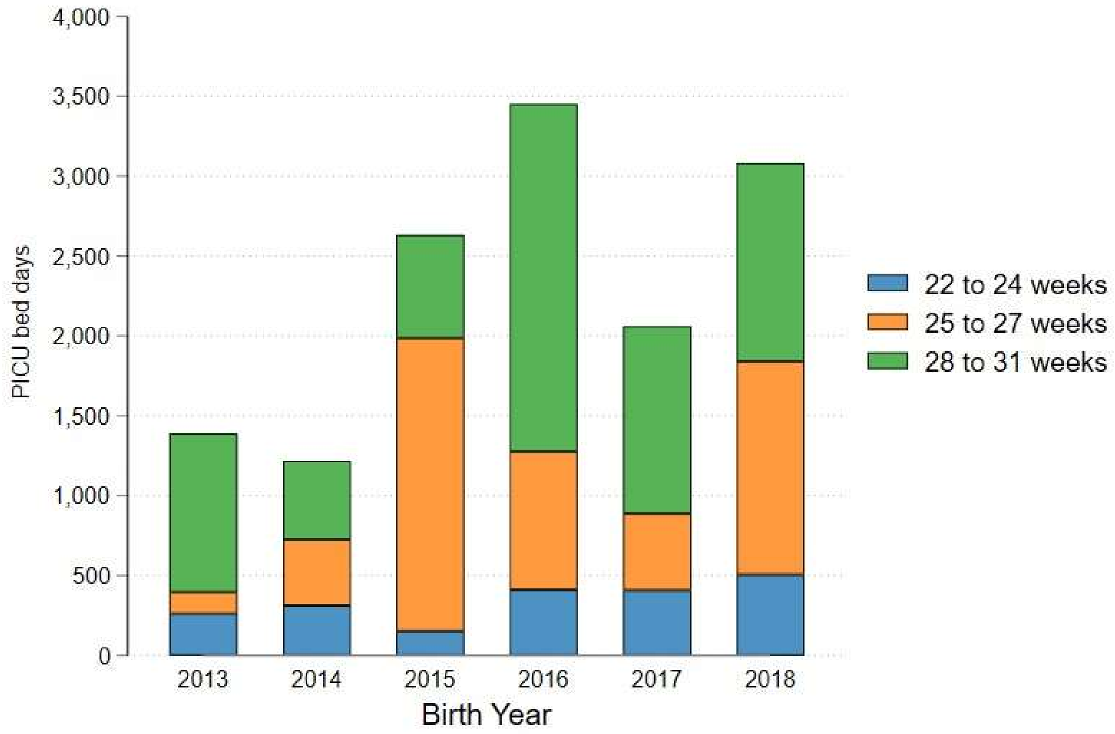

Supplementary Figure 2 – total annual PICU bed days utilised between transition from neonatal care to PICU until two years of age, by PICU admission diagnosis on transition, and birth year. Note 'Body wall' diagnoses include congenital abdominal wall defects, congenital diaphragmatic hernia, inguinal hernia, and codes for open laparotomy procedures.

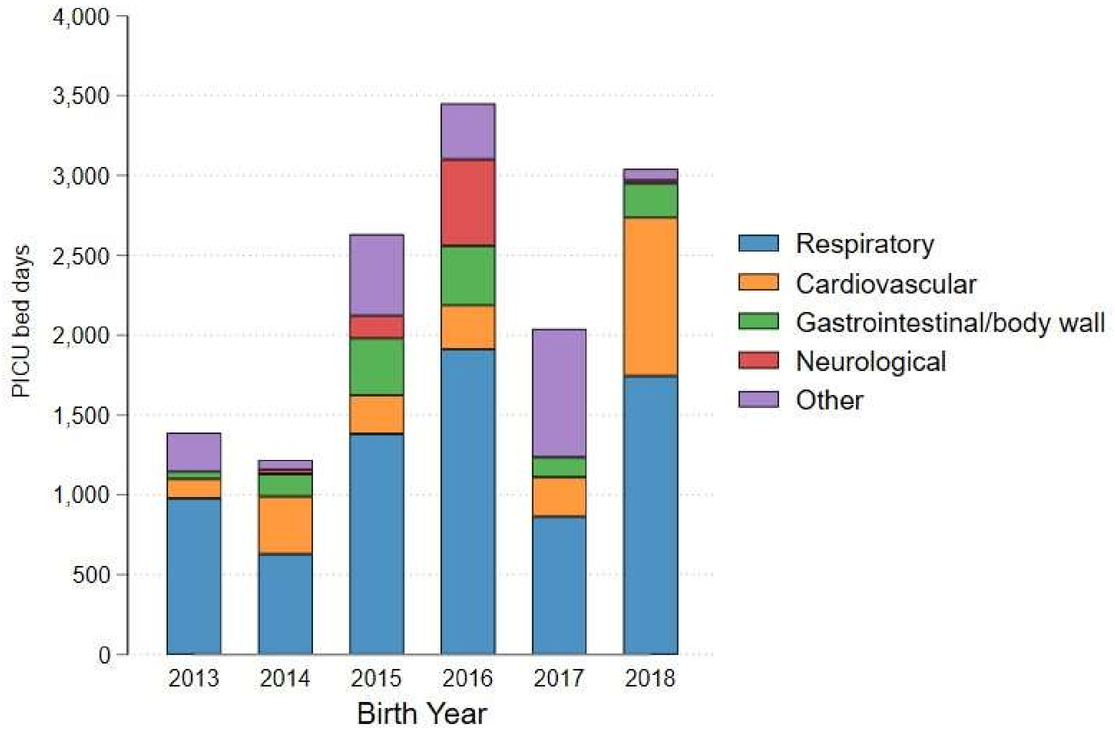

Supplementary Figure 3 – Box and whisker plot of corrected gestational age at transition to PICU, by all primary diagnoses for transition, then by primary diagnosis category. Note 'Body wall' diagnoses include congenital abdominal wall defects, congenital diaphragmatic hernia, inguinal hernia, and codes for open laparotomy procedures.

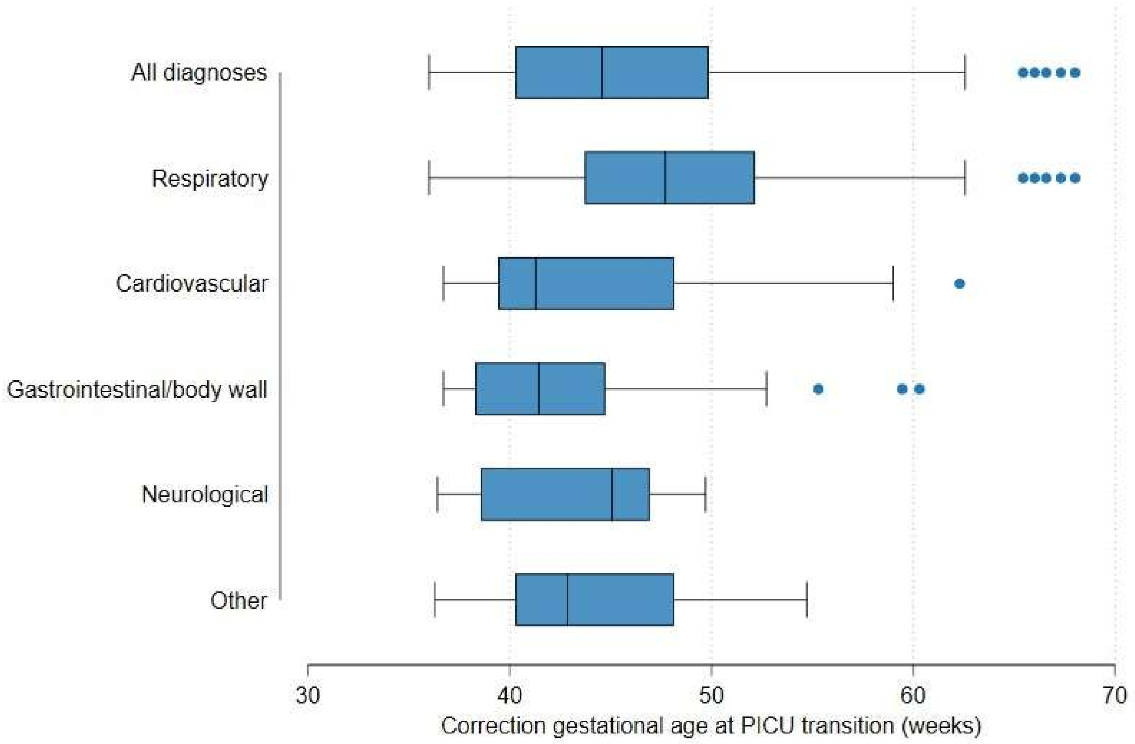

Supplement: online supplemental file 1 [file fetalneonatal-110-4-s001.pdf]
